# Biogenesis of Mitochondrial Metabolite Carriers

**DOI:** 10.3390/biom10071008

**Published:** 2020-07-07

**Authors:** Patrick Horten, Lilia Colina-Tenorio, Heike Rampelt

**Affiliations:** 1 Institute of Biochemistry and Molecular Biology, ZBMZ, Faculty of Medicine, University of Freiburg, 79104 Freiburg, Germany; patrick.horten@biochemie.uni-freiburg.de (P.H.); lilia.colina@biochemie.uni-freiburg.de (L.C.-T.); 2 Faculty of Biology, University of Freiburg, 79104 Freiburg, Germany; 3 CIBSS Centre for Integrative Biological Signalling Studies, University of Freiburg, 79104 Freiburg, Germany

**Keywords:** mitochondrial carrier, metabolite transport, mitochondrial pyruvate carrier, sideroflexin, TOM, TIM chaperones, TIM22, protein translocation, mitochondrial biogenesis

## Abstract

Metabolite carriers of the mitochondrial inner membrane are crucial for cellular physiology since mitochondria contribute essential metabolic reactions and synthesize the majority of the cellular ATP. Like almost all mitochondrial proteins, carriers have to be imported into mitochondria from the cytosol. Carrier precursors utilize a specialized translocation pathway dedicated to the biogenesis of carriers and related proteins, the carrier translocase of the inner membrane (TIM22) pathway. After recognition and import through the mitochondrial outer membrane via the translocase of the outer membrane (TOM) complex, carrier precursors are ushered through the intermembrane space by hexameric TIM chaperones and ultimately integrated into the inner membrane by the TIM22 carrier translocase. Recent advances have shed light on the mechanisms of TOM translocase and TIM chaperone function, uncovered an unexpected versatility of the machineries, and revealed novel components and functional crosstalk of the human TIM22 translocase.

## 1. Introduction

The mitochondrial inner membrane separates two aqueous compartments, the matrix and the intermembrane space, that differ in their protein and metabolite composition and host distinct metabolic pathways. The inner membrane is also the site of oxidative phosphorylation, and its integrity is crucial to maintain the electrochemical membrane potential that fuels ATP synthesis as well as mitochondrial biogenesis and function. Therefore, metabolite transport into or out of the matrix relies on carrier proteins that facilitate diffusion of specific substrates across the membrane or use the membrane potential to transport metabolites.

Most mitochondrial metabolite carriers belong to the mitochondrial carrier family (MCF, in humans SLC25 for solute carrier family 25). It comprises more than 50 members in humans and over 30 in yeast, and includes the most abundant inner membrane proteins [1,2,3,4]. MCF substrates range from nucleotides and amino acids to cofactors, intermediates of oxidative metabolism, and inorganic ions. Thus, they perform crucial functions in mitochondrial metabolism, and mutations in carrier genes are associated with a variety of human pathologies [5]. The mitochondrial pyruvate carrier (MPC) belongs to an unrelated protein family and functions as a hetero-dimer that requires both subunits for carrier activity [6,7]. The sideroflexin family constitutes a third metabolite carrier family, members of which have recently been discovered to function as serine transporters in one-carbon metabolism [8,9,10].

**Figure 1:**
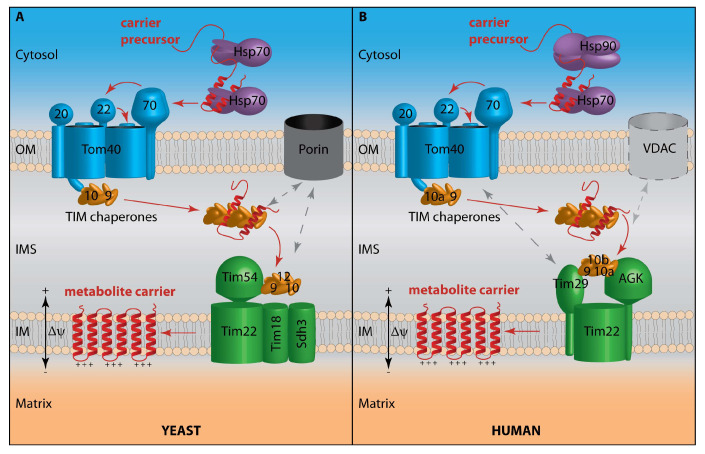
The carrier pathway in the yeast *S. cerevisiae* (**A**) and in humans (**B**) handles the recognition, translocation and membrane integration of mitochondrial metabolite carriers into the inner membrane. Carrier precursors are bound by chaperones in the cytosol and recognized at the translocase of the outer membrane (TOM) by the Tom70 receptor. After their transfer through the outer membrane, they are bound in the intermembrane space by the hexameric TIM chaperones, Tim9-Tim10 in yeast (**A**) or Tim9-Tim10a in humans (**B**). The TIM chaperones guide the precursor through the aqueous compartment to the membrane-bound TIM chaperone complex consisting of Tim9-Tim10-Tim12 in yeast (**A**) or Tim9-Tim10a-Tim10b in humans (**B**). Substrate transfer to the carrier translocase of the inner membrane (TIM22) is aided by interactions with outer membrane proteins (dashed arrows) involving the metabolite channel porin/VDAC in yeast (**A**), or the TOM complex in humans (**B**). In humans, VDAC was found in association with TIM22 components (**B**) and, thus, might participate in carrier biogenesis similarly to porin. The TIM22 carrier translocase integrates the precursors into the inner membrane in a membrane potential-dependent manner. OM, outer membrane; IMS, intermembrane space; IM, inner membrane; ∆ψ, membrane potential; Hsp70, Hsp90, cytosolic ATP-dependent chaperones; Tom40, pore-forming component of the TOM complex; Tom20, Tom22, Tom70, receptors of the TOM complex; porin/VDAC, voltage-dependent anion channel; Tim22, core component of the TIM22 translocase; Tim18, Sdh3 (succinate dehydrogenase 3), Tim54, auxiliary subunits of the yeast TIM22 translocase; Tim29, AGK (acylglycerol kinase), auxiliary subunits of the human TIM22 translocase.

Like the vast majority of mitochondrial proteins, carriers are encoded in the nuclear genome and synthesized by cytosolic ribosomes. Therefore, they have to be specifically recognized and imported into the correct mitochondrial compartment. A multitude of protein translocases cooperates in the biogenesis of proteins destined for the different mitochondrial compartments [11,12,13,14]. Most precursors of mitochondrial inner membrane proteins are imported by the presequence translocase of the inner membrane (TIM23). However, carrier precursors generally lack a presequence, although a few contain an N-terminal extension that can improve solubility and translocation across the outer membrane [15,16,17]. Instead, carriers are targeted to mitochondria by internal signals. The internal targeting signals are not well defined, and carriers apparently contain several such motifs with different properties. For their biogenesis, metabolite carriers utilize a specialized import pathway involving the carrier translocase of the inner membrane (TIM22) [12,13,17,18,19,20] (Figure 1). This pathway can be divided into consecutive, biochemically defined stages: Stages I and II take place in the cytosol and on the mitochondrial surface, leading to import of the carrier precursor by the translocase of the outer membrane (TOM). In the intermembrane space (IMS), the precursor is bound by small TIM chaperones (stage III) and handed over to the TIM22 translocase (stage IV) which integrates the carrier into the inner membrane (stage V). The membrane potential across the inner membrane provides the driving force for membrane integration: Positively charged carrier sequences are subject to an electrophoretic force that pulls them into the matrix.

**Figure 2:**
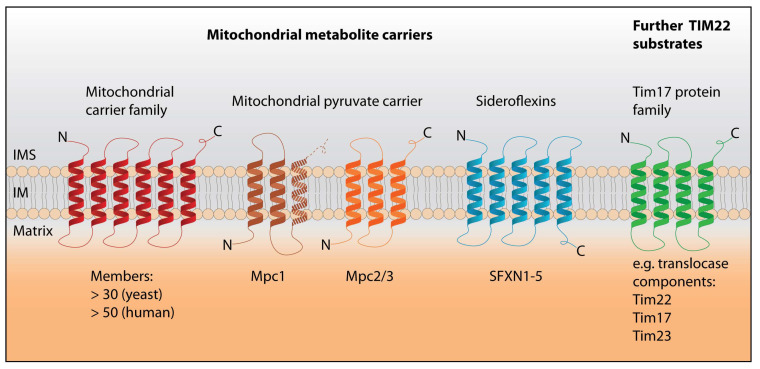
Substrates of the TIM22 carrier import pathway. Mitochondrial carriers of the mitochondrial carrier family (MCF)/SLC25 family (**red**), the components of the mitochondrial pyruvate carrier (**brown**, **orange**), as well as sideroflexins (**blue**) are imported into mitochondria via the TIM22 carrier pathway. MCF/SLC25 proteins have a uniform topology with 6 transmembrane segments [4,12,17,19,24]. In contrast, Mpc2/Mpc3 has only 3 TM segments, and Mpc1 has 2 or 3 TM segments [6,7,35,36,37]. The third unique family of metabolite carriers, the sideroflexins, has 5 TM domains with the N-terminus in the intermembrane space (IMS) [8,38,39]. Aside from metabolite carriers, the TIM22 pathway also imports the members of the Tim17 protein family including the translocase components Tim17, Tim22 and Tim23 (**green**). IMS, intermembrane space; IM, inner membrane.

Mitochondrial carriers of the MCF/SLC25 family are the eponymous substrates of the TIM22 carrier translocase pathway [12,13,17,19] (Figure 1). The best studied carrier is the ADP/ATP carrier AAC (yeast)/ANT (human; adenine nucleotide translocator) that has been employed both as a model substrate to study carrier biogenesis and for the analysis of MCF structure and transport mechanism [4,17,18,21,22,23]. Mitochondrial carriers of the MCF/SLC25 family have a tripartite organization, with three homologous repeats consisting of two transmembrane segments each. They uniformly possess six transmembrane segments and expose both their N- and C-termini to the intermembrane space (Figure 2) [4,12,17,19,24]. Mitochondrial carriers transport substrates by enabling alternate access of the substrate(s) to the matrix and the intermembrane space while maintaining membrane impermeability for non-substrates [4,25,26,27,28]. Divergent members of the MCF with a differing number of TM segments are localized in the outer membrane and have acquired functions distinct from metabolite transport [4,29,30].

Interestingly, the components of the heterodimeric mitochondrial pyruvate carrier (MPC) have recently been discovered as further substrates of the TIM22 pathway (Figure 1) [31,32]. In contrast to the classical mitochondrial carriers, they are related to sugar transporters of the eukaryotic sugars will eventually be exported transporter (SWEET) and prokaryotic semiSWEET families [33,34]. SWEET transporters possess seven TM segments that are arranged into two triple-helix bundles connected by another α-helix. SemiSWEETs instead consist of one triple-helix bundle and assemble to dimers, forming a six TM functional unit like the SWEETs. MPC subunits MPC2 (mammals) as well as Mpc2 and Mpc3 (yeast) have three TM segments (Figure 2). For MPC1/Mpc1, the topology is not entirely clear: It has been suggested that they have only two TM segments with both termini in the matrix, or alternatively that they share the same topology as Mpc2/Mpc3 [6,7,35,36,37] (Figure 2).

Additionally, recent studies indicate that the sideroflexins, with five transmembrane segments and the N-terminus in the intermembrane space (IMS), also depend on the TIM22 carrier pathway for their biogenesis [38,39] (Figure 2). Thus, the mitochondrial pyruvate carrier components and the sideroflexins with their unique topologies have challenged long-held views of the structural requirements for TIM22 substrates.

## 2. Carrier Recognition at the TOM Complex

Due to their hydrophobic nature, mitochondrial carrier precursors in the cytosol are bound by chaperones to prevent their aggregation (stage I). Since carrier import takes place post-translationally, the soluble stage can be distinguished from stage II that consists in precursor targeting to the translocase of the outer membrane (Figure 1, Table 1) [13,14,17,19]. The TOM complex is the main entry gate by which almost all precursor proteins destined to the different mitochondrial compartments gain access to the organelle [12,14,40,41]. It forms dimers *in vivo* and consists of the Tom40 β-barrel pore and six α-helical membrane proteins: Tom5, Tom6, Tom7 as well as the receptors Tom22, Tom20 and Tom70 [42,43,44]. Aggregation of the highly hydrophobic carrier precursors in the cytosol is prevented by molecular chaperones. In yeast, carriers are chaperoned mainly by Hsp70, whereas in mammalian cells both Hsp70 and Hsp90 participate in carrier biogenesis, along with several co-chaperones [14,45,46,47,48,49]. Recognition of precursors at the TOM complex is mediated by the receptors Tom20 and Tom70. They can functionally substitute for each other sufficiently well for single deletions to be viable, however Tom20 preferentially recognizes precursors that contain a presequence, while Tom70 preferentially binds precursors with internal targeting sequences including carrier proteins such as the ADP/ATP carrier or the phosphate carrier [12,13,49,50,51,52,53,54,55,56,57,58,59,60]. Tom70 not only interacts with the precursor, but also with the associated chaperone(s) via tetratricopeptide repeats (TPR) that bind the C-termini of Hsp70 or Hsp90 chaperones [14,46,49,61]. Moreover, one tripartite carrier precursor of the MCF/SLC25 family can recruit three Tom70 dimers, with each of the repeats participating in the interaction [21]. Thus, the interactions of Tom70 with the precursor-chaperone complex likely contribute to prevention of its aggregation. Import of carriers can be stalled at stage II by depletion of ATP (Table 1). ATP binding to Hsp70 triggers substrate release from Hsp70, the carrier precursor is handed over to the central receptor Tom22, and individual helix-loop-helix modules are threaded into the Tom40 pore in a hairpin-like conformation [21,62]. It is currently unclear how mitochondrial pyruvate carrier precursors with their distinct topology are handled by the TOM complex, although it is tempting to speculate that at least the two C-terminal TM segments of Mpc2/Mpc3 may be recognized in a fashion similar to classical carriers. Interestingly, even during translocation through the TOM complex, carriers follow a different route than presequence precursors, involving the distal regions of the Tom40 dimer and the N-terminal extension of Tom40 [42,44]. It was proposed that translocation through TOM is aided by interaction of positively charged regions in the precursors with the negatively charged inner surface of the Tom40 β-barrel [63], which is consistent with the previously reported head-first insertion of helix-loop-helix modules by their positively charged loops [21].

The efficiency of carrier recognition at the TOM complex is subject to metabolic regulation. Tom70 is phosphorylated by protein kinase A specifically during non-respiratory growth of yeast on glucose, resulting in an impaired interaction with Hsp70 and concomitantly reduced carrier import [64]. Thus, the efficiency of carrier biogenesis can be adjusted to the metabolic requirements of respiratory versus non-respiratory growth.

## 3. En Route through the Intermembrane Space

Unlike the TIM23 translocase that imports presequence proteins into the matrix or the inner membrane, the translocation of carrier precursors through the TOM complex is apparently not tightly coupled to integration into the inner membrane by the TIM22 carrier translocase [12,17,65,66]. Instead, once a carrier precursor has traversed the Tom40 pore and reached the IMS, it is bound by small TIM chaperones to prevent aggregation during its transit through the aqueous IMS environment to the inner membrane (stage III) [67,68,69,70,71,72,73] (Figure 1, Table 1). Translocation through the TOM complex is coupled to TIM chaperone binding [71]. The small TIM chaperones form ring-like hetero-hexameric complexes that bind carriers in an extended conformation [73,74,75]. The predominant TIM chaperone complex consists of alternating Tim9 and Tim10 subunits (Tim9 and Tim10a in humans) and is required for carrier import; the alternative Tim8-Tim13 complex (Tim8a or Tim8b and Tim13 in humans) has partially redundant substrate specificity [73,75,76,77,78,79]. Mutations in Tim8a cause the deafness–dystonia syndrome called Mohr–Tranebjærg syndrome [80], however, novel evidence suggests that the underlying molecular mechanism reflects a new function of Tim8a in cytochrome c oxidase maturation rather than a defective TIM22 carrier pathway [79]. The small TIM chaperones interact with the N-terminal extension of Tom40 that participates in carrier translocation, so they are ideally positioned to receive their cargo from the TOM complex [21,42,44,71]. Stage III of carrier import can be further subdivided, where stage IIIa denotes carriers bound to soluble TIM chaperones that may be associated with TOM or soluble in the IMS (Table 1) [17,19]. A recent comprehensive study demonstrated that an MCF carrier with six TM segments is bound by two TIM chaperone hexamers, and the precursor is chaperoned by interacting with a conserved hydrophobic cleft between the two tentacle-like α-helices of the small TIM proteins [73]. This conserved substrate binding region is also required for chaperoning of the structurally unrelated mitochondrial pyruvate carrier [31]. The soluble TIM complexes transfer carrier precursors to a separate TIM chaperone complex that is associated with the TIM22 carrier translocase of the inner membrane. This membrane-bound TIM hexamer consists of Tim9/Tim10/Tim12 (yeast) or Tim9/Tim10a/Tim10b (human) [67,81,82,83,84,85,86] (Figure 1). Carrier precursors bound to the membrane-associated TIM chaperones represent stage IIIb of the carrier import pathway where the precursor is tethered to the inner membrane, but not yet inserted (Table 1) [17,19]. Since this is the last step that is independent of the membrane potential ∆ψ, precursors accumulate in stage III upon dissipation of the membrane potential. The dependence of carrier import on TIM chaperones also allows to experimentally distinguish this import pathway into the inner membrane from the presequence pathway where import into a protease-protected environment depends on ∆ψ due to the coupling of TOM and TIM23 [65,66].

Unexpectedly, transport of carriers to the TIM22 carrier translocase is also aided by porin/voltage-dependent anion channel (VDAC), the major metabolite channel of the outer membrane (Figure 1) [87,88,89,90]. In addition to interacting with TIM chaperones as well as carrier precursors, porin recruits the TIM22 translocase and thereby brings the outer and inner membrane in close proximity, which may enhance carrier biogenesis [88,89]. Since porin, unlike the protein translocases, is significantly upregulated upon respiratory growth [91], this novel physiological role may support the higher levels of protein import required for metabolic remodeling of the mitochondria. While a contribution of VDACs to carrier biogenesis in human mitochondria has not been studied directly, they were found to interact with TIM22 [92,93].

## 4. Membrane Integration by the TIM22 Carrier Translocase

The TIM22 translocase consists of the Tim22 protein, several auxiliary subunits that have roles in assembly and stabilization of the complex, and TIM chaperones. In yeast, the additional subunits comprise Tim54, Sdh3, which also interacts with Sdh4 as part of the succinate dehydrogenase complex, and Tim18, a homolog of Sdh4 [12,17,19,20,94,95,96,97,98,99,100] (Figure 1A). Until recently, the only known membrane integral TIM22 component in humans was Tim22 itself, however, several studies have discovered two new subunits (Figure 1B). The metazoan-specific subunit Tim29, which like Tim54 is exposed to the IMS and can be crosslinked to TIM chaperones, is required for TIM22 assembly and efficient import of some substrates [92,101]. Moreover, Tim29 interacts with Tom40, indicating that there may be coupling between TOM and TIM22 in human cells [92], in contrast to yeast. The most recently identified subunit of human TIM22 is acylglycerol kinase (AGK) that phosphorylates glycerides to generate lysophosphatidic acid or phosphatidic acid [102,103,104]. AGK has a dual role in human mitochondria: It is required for TIM22 stability and carrier import independently of its lipid kinase activity, and loss of AGK results in TCA (tricarboxylic acid) cycle defects; however, kinase deficiency causes aberrant mitochondrial ultrastructure and concomitantly reduced respiration [103,104]. Mutations in AGK cause the mitochondrial disease Sengers syndrome [105,106], and its novel role as part of the TIM22 translocase appears to account for the disease phenotype [103]. Aside from AGK, mutations in Tim22 that impair carrier import were also recently reported to result in human pathology with neuromuscular defects [32,107]. Interestingly, human TIM22 interacts with the mitochondrial contact site and cristae organizing system (MICOS) [93], an inner membrane protein complex that is crucial for native cristae architecture and that forms contact sites between the two mitochondrial membranes [108,109,110]. Upon MICOS disruption, carrier import is specifically impaired, indicating that MICOS-mediated membrane contact sites might support efficient carrier biogenesis in human cells [93].

The TIM22 complex is a voltage-gated preprotein translocase that is thought to insert one helix—matrix loop—helix repeat at a time in a hairpin conformation into the inner membrane [19,20,86,97]. At least a low membrane potential is required for transfer of a precursor from the TIM chaperones to TIM22. Precursors can be trapped experimentally at this stage IV by reducing the membrane potential with ionophores (Table 1) [20]. In the presence of a higher membrane potential as well as of an internal targeting sequence, TIM22 integrates the protein into the inner membrane by an unknown mechanism involving lateral release, and the carrier reaches the mature stage V [20].

The modular topology of mitochondrial carriers of the MCF/SLC25 family—with repeats of helix-loop-helix domains, the termini facing the IMS and positively charged loops in the matrix—was long assumed to be a requirement for substrates of the TIM22 carrier pathway. This pathway also imports members of the Tim17 protein family that includes Tim22 itself as well as the TIM23 translocase components Tim23 and Tim17, all of which have four TM segments but otherwise share the topology of classical carriers [73,75,76,77,111,112,113] (Figure 2). As mentioned, both TOM and TIM22 translocases act on paired helices during import of classical carriers, including the dicarboxylate carrier, the ADP/ATP carrier and the phosphate carrier, as well as during import of Tim22-related proteins [21,70,77,114,115,116], in contrast to the linear import of other mitochondrial proteins. Until recently, multi-spanning inner membrane proteins apart from the MCF and Tim17 families were thought to be imported into mitochondria by the other translocases of the inner membrane: By the TIM23 translocase [11,12,13,65,66,117], by the oxidase assembly (OXA) translocase that is responsible for the membrane integration of mitochondrially encoded proteins [118,119] or by a combination of both machineries [120,121,122]. Moreover, truncated variants of carrier proteins are no longer recognized as substrates of the TIM22 pathway and instead are imported by TIM23 or remain in the intermembrane space [115,116]. However, recent work has identified the mitochondrial pyruvate carrier proteins with their divergent topology as substrates of the TIM22 pathway [31,32]. What is more, the unrelated sideroflexins also rely on the TIM22 carrier translocase for their biogenesis [38,39]. Of the MPC components, at least Mpc2 and Mpc3 have unpaired TM helices and none of the MPC proteins possess more than three TM segments [6,7,35,36,37], while the sideroflexins also have an uneven number of TM segments and yet another topology [8,39] (Figure 2). Thus, multiple recent studies have revealed a surprising versatility of the TIM22 pathway. The mechanistic differences in the handling of substrates with 4 or 6 TM segments (classical TIM22 substrates) versus 2/3 (MPC subunits) or 5 TM segments (sideroflexins) are still unclear. The C-terminal helix-loop-helix domain of Mpc2 and Mpc3 might conceivably be treated similarly as a typical carrier repeat, since they share the topology and positively charged matrix loop. In addition, MPC subunits were reported to have an N-terminal α-helix whose function is unknown [37]. For the human TIM22 translocase a differential requirement of the auxiliary subunits for different substrate classes has been reported: AGK is required for efficient carrier import and dispensable for the import of Tim22 and related proteins [103,104], whereas the opposite is the case for Tim29 [92]. Interestingly, sideroflexins, like classical carriers, rely on AGK, while Tim29 is dispensable [38]. It will be very interesting to learn how TIM22 adapts its function to import this range of structurally distinct substrates.

## 5. Perspectives

The biogenesis of mitochondrial metabolite carriers is still not fully understood despite the fact that the TIM22 import pathway has been under scientific investigation for decades. The recent discovery of two human TIM22 components and the very limited insight into the mechanism of membrane integration by the carrier translocase exemplify how much fundamental information is still lacking. Novel findings indicate that carrier import may benefit from contact sites between the outer and inner membranes after all. Moreover, the TIM22 pathway has turned out to be unexpectedly versatile regarding its substrate requirements. Finally, it seems likely that the biogenesis of proteins with such a central role in mitochondrial physiology is regulated at different steps. While there is precedent for this notion from yeast, the human TIM22 pathway still awaits characterization of regulatory factors. Thus, the biogenesis of mitochondrial metabolite carriers remains an exciting field of study that is expected to generate important insights into mitochondrial physiology.

## Figures and Tables

**Table 1:** Stages of carrier biogenesis via the TIM22 carrier import pathway.

TIM22 Carrier Import Pathway	Characteristics of Individual Stages in Carrier Biogenesis
Stage I	The carrier precursor is bound to cytosolic chaperones upon its synthesis, forming a soluble complex not associated with mitochondria.
Stage II	The precursor-chaperone complex is recognized by the Tom70 receptor of the TOM complex and can be arrested on the mitochondrial surface by ATP depletion. ATP binding triggers dissociation of Hsp70 chaperones and progression of the precursor.
Stage III IIIa:	The carrier precursor is translocated through the TOM complex into the IMS and concomitantly bound by soluble TIM chaperones (mainly Tim9-Tim10 in yeast, Tim9-Tim10a in humans).
IIIb:	The carrier precursor is handed over to the TIM22-bound TIM chaperones (Tim9-Tim10-Tim12 in yeast, Tim9-Tim10a-Tim10b in humans), resulting in tethering to the inner membrane.
Stage IV	In the presence of a low membrane potential, the carrier precursor is transferred to TIM22 and inserted into the inner membrane as a docked precursor.
Stage V	Formation of the mature, inner-membrane integrated carrier and release from TIM22 requires the presence of a higher membrane potential.
